# HYPEST study: profile of hypertensive patients in Estonia

**DOI:** 10.1186/1471-2261-11-55

**Published:** 2011-08-31

**Authors:** Elin Org, Gudrun Veldre, Margus Viigimaa, Peeter Juhanson, Margus Putku, Mai Rosenberg, Kärt Tomberg, Tiina Uuetoa, Maris Laan

**Affiliations:** 1 Human Molecular Genetics Research Group, Institute of Molecular and Cell Biology, University of Tartu, Tartu, Estonia; 2 Department of Cardiology, University of Tartu, Tartu, Estonia; 3 Tallinn University of Technology, Institute of Biomedical Engineering, Tallinn, Estonia; 4 Centre of Cardiology, North Estonia Medical Centre, Tallinn, Estonia; 5 Department of Internal Medicine, University of Tartu, Tartu, Estonia; 6 Centre of Cardiology of Clinics of Internal Medicine, East Tallinn Clinicum, Tallinn, Estonia; 7 Department of Human Genetics, University of Michigan, Ann Arbor, MI, USA

## Abstract

**Background:**

More than one third of adult population in Estonia has problems with elevated blood pressure (BP). The *Hypertension in Estonia *(HYPEST) study represents the country's first hypertension-targeted sample collection aiming to examine the epidemiological and genetic determinants for hypertension (HTN) and related cardiovascular diseases (CVD) in Estonian population. The HYPEST subjects (n = 1,966) were recruited across Estonia between 2004-2007 including clinically diagnosed HTN cases and population-based controls. The present report is focused on the clinical and epidemiological profile of HYPEST cases, and gender-specific effects on the pathophysiology of hypertension.

**Methods:**

Current analysis was performed on 1,007 clinically diagnosed HTN patients (617 women and 390 men) aged 18-85 years. The hypertensives were recruited to the study by BP specialists at the North Estonia Medical Center, Centre of Cardiology, Tallinn or at the Cardiology Clinic, Tartu University Hospital, Estonia. Longitudinal BP data was extracted retrospectively from clinical records. Current and retrospective data of patient's medical history, medication intake and lifestyle habits were derived from self-administrated questionnaire and each variable was examined separately for men and women. Eleven biochemical parameters were measured from fasting serum samples of 756 patients.

**Results:**

The distribution of recruited men and women was 39% and 61% respectively. Majority of Estonian HTN patients (85%) were overweight (BMI ≥ 25 kg/m^2^) and a total of 79% of patients had additional complications with cardiovascular system. In men, the hypertension started almost 5 years earlier than in women (40.5 ± 14.5 vs 46.1 ± 12.7 years), which led to earlier age of first myocardial infarction (MI) and overall higher incidence rate of MI among male patients (men 21.2%, women 8.9%, *P *< 0.0001). Heart arrhythmia, thyroid diseases, renal tubulo-intestinal diseases and hyperlipidemia were more prevalent in hypertensive women compared to men (*P *< 0.0001). An earlier age of HTN onset was significantly associated with smoking (*P *= 0.00007), obesity (BMI ≥ 30 kg/m^2^; *P *= 0.0003), increased stress (*P *= 0.0003) and alcohol consumption (*P *= 0.004).

**Conclusion:**

Understanding the clinical profile of HTN patients contributes to CVD management. Estonian hypertension patients exhibited different disease and risk profiles of male and female patients. This well-characterized sample set provides a good resource for studying hypertension and other cardiovascular phenotypes.

## Background

Hypertension is defined as a physiological condition characterized by consistently elevated blood pressure (BP). High BP affects approximately 20 - 30% of the adult population in modern societies and its prevalence has been predicted to increase as high as 60% in 2025 [1,2]. Among European countries Estonia stands out with high prevalence of hypertension, affecting more than one third of adult population [3]. As high BP is a major risk factor for cardiovascular morbidity and mortality [4], its early diagnosis and timely treatment is crucial for postponing and preventing cardiovascular diseases. In 2005, the mortality from cardiovascular disease among Estonian men aged < 65 years was three times higher than the average of European Union member states [3]. The proportion of hypertensive population in Estonia is increasing hand-in-hand with its economic status, consistent with studies showing that introducing the westernized life style contributes to generally elevated BP levels [1,5]. Despite major public health problems caused by hypertension, only limited data are available on its epidemiology, etiology and risk factors in Estonian population [6,7]. To provide a better understanding of lifestyle, environmental and genetic risk factors leading to elevated BP in Estonian population, we have established the country's first hypertension-targeted sample collection HYPEST (*HYPertension in ESTonia*).

There is an increasing body of data showing gender-dependent effects of commonly accepted confounders (such as age, lifestyle factors or genetic susceptibility) contributing to the determination of an individual's BP [8-12]. For instance, obesity has been shown to be predominant risk factor for women [11], whereas smoking enhances the development of hypertension in men [12]. Current report evaluates gender-specific pathophysiology and risk factors of hypertension in Estonia by comparing disease and lifestyle profiles of male and female essential hypertension patients recruited in the framework of the HYPEST study.

## Methods

### Study design and recruitment of HYPEST participants

The HYPEST (*HYPertension in ESTonia*) study has been approved by the Ethics Committee on Human Research of University of Tartu (no. 122/13, 22.12.2003; 137/20, 25.04.2005). The study was carried out in compliance with the Helsinki Declaration and all the participants have given their written informed consent. The HYPEST sample collections have been recruited to target the genetic-epidemiological component of essential hypertension and cardiovascular disease in Estonian population. The subjects (n = 1966; age range 18-85 years) were recruited across Estonia between 2004-2007 including (i) patients with clinically diagnosed essential hypertension (HTN) (n = 1007) and (ii) a population controls of long-term blood donors (n = 959). All of the study participants are of Eastern European ancestry. Blood samples for DNA extraction and further genetic analysis were obtained from 1,823 HYPEST individuals (n = 864 HTN cases; n = 959 population controls). The genomic DNA of HYPEST participants are stored at -80°C at the laboratory of the Human molecular genetics research group (headed by Maris Laan), Institute of Molecular and Cell Biology, University of Tartu. Current report is only focused on the epidemiology of HYPEST patients with clinically diagnosed HTN.

**Figure 1:**
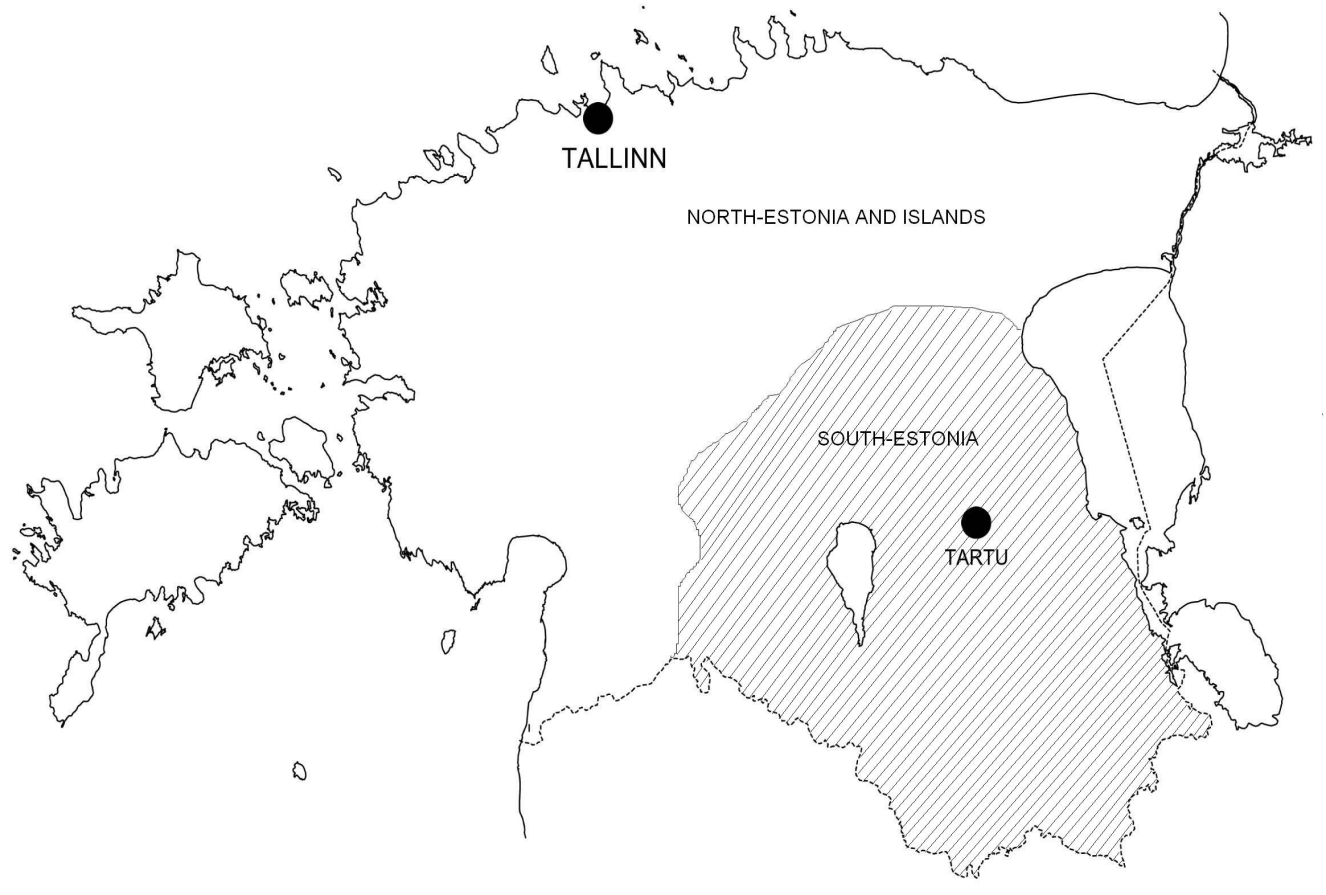
Physical map of Estonia (~45000 km^2^) and two recruitment centers: North-Estonian Regional Hospital, Tallinn representing Northern, North-Eastern, South-Western and Western (including largest islands) parts of Estonia; University of Tartu Clinics, Tartu, representing Central, South-Eastern and Southern parts of Estonia.

Estonian HTN patients (n = 1,007) were recruited by blood pressure (BP) specialists during patients' ambulatory visits or hospitalization at the North Estonia Medical Center, Centre of Cardiology, Tallinn or at the Cardiology Clinic, Tartu University Hospital, Estonia. These two main healthcare centers cover major part of Estonia with premium medical aid (Figure 1). Alternatively, the patients with clinically diagnosed long-term HTN were invited to the study by the personnel of cardiology centres based on subject's cardiovascular health records at the clinic. Diagnosis was based on the following International Classification of Diseases 10^th ^Revision (ICD-10) codes: essential (primary) hypertension (I10), hypertensive heart disease with (congestive) heart failure (I11.0) and/or hypertensive heart disease without (congestive) heart failure I11.9).

### Collection of blood pressure and epidemiological data

Longitudinal data of systolic and diastolic blood pressure (BP) readings (before and during antihypertensive treatment) of the HYPEST HTN patients were extracted retrospectively from clinical records. The average of each participant BP readings was used for the analyses in the current study. Blood pressure measures had been taken after a rest in sitting position using a conventional mercury column sphygmomanometer and size adjusted cuffs, and the procedures were performed by trained clinicians following the standardized protocol [13]. Hypertension was defined as a systolic BP (SBP) ≥ 140 mm Hg and/or diastolic BP (DBP) ≥ 90 mm Hg during the healthcare centre visit. Subjects were asked if they had had treatment or were being treated currently with antihypertensive drugs, and the details on used anti-hypertensive drugs were collected.

The HYPEST study participants filled out a self-administered epidemiological questionnaire recording their past and present health and lifestyle. The questionnaire included participants' general demographic data (age, sex, nationality, place of residence), lifestyle factors (tobacco and alcohol consumption, preferences of food consumption), physical activity, stress, health status and medical records of the subject. Additionally, self-reported familiar disposition to cardiovascular disease was recorded. Personal medication, including past and current prescription and over-the-counter drugs were collected based on self-reported data and available clinical records. Additionally, for female participants' data regarding their reproductive history (number of children, pregnancy complications and hormonal replacement therapy) was collected. Information about smoking status included the previous and current smoking status as well as the amount of tobacco smoked (number of cigarettes, cigarillos, cigars or pipes), age of beginning and quitting (for former smokers). Data on alcohol consumption habits during lifetime included the drinking status and the volume of consumed alcohol (wine, beer and spirit) during the week. Body mass index was based on participant's body weight and height values during recruitment and was defined as weight/height^2^. Additionally, self-reported data were documented for birth weight, birth height and weight at age 18 years. Physiological stress was rated based on self-reported information: (i) no stress or occasional short stress episodes during their life; (ii) exposure to regular stress.

### Analysis of serum biomarkers

Among the enrolled HTN patients (n = 1,106) 756 individuals (484 women/272 men) agreed to provide blood samples for serum biomarker analysis. The venous blood samples for clinical chemistry analysis were drawn in the morning after an overnight fast. Clinical chemistry assays were performed using standardized assays (Cobas Integra 800^® ^analytical platform, Roche Diagnostics, Inc. USA) applied at the accredited hospital laboratories in Tartu (United Laboratories, Tartu University Clinics) and in Tallinn (Diagnostic Division Laboratory, North Estonia Medical Center). The following parameters from serum were estimated: (1) Sodium (S-Na) [mmol/l], (2) Potassium (S-K) [mmol/l], (3) Urea (fS-Urea) [mmol/l], (4) Creatinine (fS-Crea) [μmol/l], (5) Uric acid (S-UA) [μmol/l], (6) Albumin (S-Alb) [g/l], (7) Cholesterol (fS-Chol) [g/l], (8) Triglycerides (fS-Trigl) [mmol/l], (9) HDL-Cholesterol (fS-HDL-C) [g/l], (10) LDL-Cholesterol (fS-LDL-C) [g/l] and (11) C-reactive protein (fS-CRP) [mg/L]. The tests of the serum biomarkers have been accredited by Estonian Accreditation Center [14] according to the standards of the European co-operation for accreditation [15]. Measurement uncertainty was estimated using EURACHEM guidelines [16]. Measurement uncertainty is defined as the parameter associated with the result of a measurement that characterizes the dispersion of the values that could reasonably be attributed to the measuring (e.g. the concentration of a biomarker). Additional aliquots were stored at -80°C.

### Statistical analysis

Statistical analysis was performed using Statistical Analysis System (SAS) software (version 9.1) (SAS Institute Inc., Cary, North Carolina, USA). Variables were estimated separately for men and women, and significance of gender differences was compared using Mann-Whitney U-test (two-sided) for quantitative (not normally distributed) and categorical variables. Gender comparison of SBP and DBP without and under antihypertensive treatment was performed by analysis of covariance (ANCOVA) adjusted by median age of each patient at BP readings during pre-treatment or medication period, respectively. Fisher's exact test was used for calculating of frequency differences. Statistical significance was assessed for *p *< 0.05.

## Results

### Recruitment and demographic data of the study population

Estonian patients with clinically diagnosed essential hypertension (HTN) were invited to the *HYPertension in ESTonia *(HYPEST) study by the cardiology centers at the University of Tartu Clinics, Tartu and North-Estonian Regional Hospital, Tallinn. Detailed information on recruitment stages and response rates is provided in Table 1. Among 2,383 invited patients with clinically diagnosed HTN (men, n = 977; women, n = 1406), 1,106 subjects (46.4%; men, n = 424; women, n = 682) gave written informed consent. The response rate among hypertensive women was slightly higher compared to male patients (48.5% *vs*. 43.4%, respectively). Eventually, 1,007 HTN patients returned filled self-administrated epidemiological questionnaires and 756 of them (75.1%) also agreed to provide blood samples for serum biomarker analysis. The recruited Estonian HTN patients covered a wide range of age groups (18-85 years; mean 57.22 ± 11.15 years; Table 2).

**Table 1:** Recruitment strategy and efficacy of hypertension patients across Estonia

	North-Estonian Regional Hospital^a^			University of Tartu Clinics^b^			All		
	
Recruitment stage	Invited (n)	Agreed (n)	Responded (%)	Invited (n)	Agreed (n)	Responded (%)	Invited (n)	Agreed (n)	Responded (%)
Information and consent letters									
total	1221	590	48.3	1162	516	44.4	2383	1106	46.4
men	509	251	49.3	468	172	36.8	977	424	43.4
women	712	339	47.6	694	344	49.6	1406	682	48.5
Epidemiological questionnaires									
total	590	510	86.4	516	497	96.3	1106	1007	91.1
men	251	232	92.4	172	160	93.0	424	390	92.0
women	339	278	82.0	344	337	98.0	682	617	90.5
Blood serum samples									
total	510	398	78.0	497	358	72.0	1007	756	75.1
men	232	174	75.0	160	100	62.5	390	273	70.0
women	278	222	80.6	337	261	77.4	617	484	78.4

^a ^Regional coverage: Northern, North-Eastern, South-Western (including the largest island Saaremaa) of Estonia (Figure 1); ^b ^Regional coverage: Central, South-Eastern and Southern- Estonia (Figure 1); n - number of subject.

**Table 2:** Number, age- and gender-specific distribution of recruited patients with clinically diagnosed hypertension in HYPEST study

Age groups (years)	All HTN patients (%)	Men (%)	Women (%)
< 30	10 (1.0)	8 (2.1)	2 (0.3)
30-39	43 (4.3)	22 (5.8)	21 (3.4)
40-49	152 (15.1)	81 (20.8)	71 (11.5)
50-59	375 (37.2)	157 (40.3)	218 (35.3)
60-69	325 (32.3)	82 (21.0)	243 (39.4)
≥ 70	102 (10.1)	40 (10.3)	62 (10.0)
			
Total recruited	1,007	390	617
Mean age at recruitment (± SD)	57.2 (± 11.2)	54.5 (± 11.2)	58.9 (± 9.6)

HTN - hypertension; SD - standard deviation.

The final distribution of genders among HYPEST cases with self-administrated epidemiological data was 617 women/390 men (61.3%/38.7%). At the recruitment, an average female patient was 4 years older than a participating male patient (58.9 ± 9.6 *vs*. 54.5 ± 11.2 years; Table 2). Expected significant differences between men and women were detected for baseline anthropometric characteristics (mean height 177.6 *vs*.162.9 cm; mean weight 94.2 *vs*. 80.6 kg, respectively) and for self-reported birth height and weight (Table 3). Majority of Estonian HTN patents (85%) were classified as overweight (BMI ≥ 25 kg/m^2^), mean BMI was estimated as high as 30.4 ± 5.5 kg/m^2 ^for women and 29.8 ± 5.0 kg/m^2 ^for men.

**Table 3:** Gender differences in anthropometric variables among Estonian hypertension patients

	Male		Female		
		
Variable	Mean ± SD (95% CI)	Median	Mean ± SD (95% CI)	Median	*P*-value^e^ for gender comparison
Height (cm)	177.6 ± 7.4 (176.8-178.3)	178	162.9 ± 5.7 (162.5-163.4)	163	**< 0.0001**
Weight (kg)	94.2 ± 17.7 (92.4-96.0)	92	80.6 ± 15.5 (79.4-81.8)	80	**< 0.0001**
BMI (kg/m^2^)	29.8 ± 5.0 (29.3 ± 30.3)	29.1	30.4 ± 5.5 (29.9-30.8)	29.7	0.12
Birth height ^a ^(cm)	51.4 ± 1.9 (51.0-51.7)	51	50.4 ± 2.5 (50.0-50.8)	50	**< 0.0001**
Birth weight^b^ (g)	3680.4 ± 572.2 (3583.0-3777.9)	3600	3411.4 ± 650.6 (3321.9-3501.0)	3500	**< 0.0001**
Weight at 18 years ^c ^(kg)	70.7 ± 10.0 (69.6-71.9)	70	56.8 ± 8.4 (56.0-57.5)	56	**< 0.0001**

^a ^n = 98 men; n = 139 female; ^b ^n = 135 men; n = 205 female; ^c ^n = 302 men; n = 487 female;

^e ^Mann-Whitney U test; SD - standard deviation; CI - confidence interval; Highly significant *P*-values (< 0.0001) have been indicated in bold.

### Diagnosis and treatment of hypertension

At the recruitment, the mean duration of hypertension among in HYPEST male and female HTN patients was similar, 13.9 ± 12.5 and 13.1 ± 10.8 years, respectively (Table 4). However, the male patients had been on average ~5 years younger compared to hypertensive women at the clinical diagnosis of hypertension (40.5 ± 14.5 *vs*. 46.1 ± 12.7 years respectively, *P *< 0.0001). Retrospective BP readings (range 1-29 measurements/subject) had been documented for 708 HTN patients (248 men/460 women) during mean 2.3 years (range 1-17 years). BP values during antihypertensive treatment period were available for 528 (182 men/346 women) and the pre-diagnosis (without treatment) BP values were available for 344 patients (124 men/220 women). For 272 patients (101 men/171 women) both, pre-treatment and under medication BP values had been clinically recorded. BP records under antihypertensive treatment had been documented with ≥ 3 readings/subject for 328 patients and ≤ 2 readings/subject for 200 patients (mean 3.49 ± s.e.m. 0.11; median 3.0), whereas majority of available pre-treatment data (82% of patients) was represented by up to two BP readings/subject (mean 1.81 ± s.e.m. 0.01; median 1.0).

**Table 4:** Hypertension diagnosis and profile of HYPEST patients

	Male		Female		
		
	Mean ± SD (95% CI)	Median	Mean ± SD (95% CI)	Median	*P*-value for gender comparison
*Diagnosis of hypertension (HTN)*					
Diagnosis age (y)	40.5 ± 14.5 (38.8-42.2)	43	46.1 ± 12.7 (44.9-47.2)	48	< 0.0001^c^
SBP (mmHg) without treatment^a^	156.5 ± 19.9 (152.9-160.0)	155	160.1 ± 21.2 (157.2-162.9)	158	0.28^d^
DBP (mmHg) without treatment^a^	97.2 ± 13.4 (94.8-99.6)	99.5	95.1 ± 12.8 (93.4-96.8)	94	0.43^d^
*Hypertension profile at recruitment into HYPEST study*					
Duration of hypertension (y)	13.9 ± 12.5 (12.4-15.4)	9	13.1 ± 10.8 (12.2-14.1)	10	0.92^c^
SBP (mmHg) under treatment^b^	140.5 ± 17.3 (138.0-143.1)	140	144.6 ± 18.7 (142.6-146.6)	142	0.06^d^
DBP (mmHg) under treatment^b^	86.6 ± 11.0 (85.0-88.2)	85	87.4 ± 10.6 (86.3-88.5)	87.5	0.28^d^

^a ^BP determined at diagnosis of HTN before prescription of antihypertensive medication, n = 344 (124 men/220 women); ^b ^HTN patients with antihypertensive treatment n = 528 (182 men/346 women); ^c ^Mann-Whitney U test; ^d ^age-adjusted Analysis of covariance (ANCOVA); SD - standard deviation; CI - confidence interval

The mean baseline systolic (SBP) and diastolic blood pressure (DBP) of recruited patients regardless their antihypertensive treatment status, was 143.2 mmHg (range 131.8 - 156.7 mmHg) and 87.1 mmHg (range 80.3 - 94.6 mmHg), respectively. Recruited female patients exhibited a trend for higher SBP compared to men (144.6 ± 18.7 mmHg *vs*. 140.5 ± 17.3 mmHg; *P *= 0.06; Table 4). Since BP has a tendency to rise with the age, the observed differences among sexes may reflect older age of female compared to male patients (Table 2). Alternatively, this may result from the lower efficacy of BP lowering treatment among Estonian women compared to men, described previously in a report of the Estonian Society of Hypertension [7].

Among the enrolled Estonian HTN patients (n = 1,007) 588 individuals were knowledgeable about the details of their prescribed antihypertensive treatment. Majority of them (93%) was currently taking anti-hypertensive medication, 7% had temporarily quitted medication. Across all recruited HTN patients the mean period of antihypertensive treatment was 42.5 ± 40.6 months. The number of different prescribed medicines per patient during the treatment period was as high as 3.4 ± 1.7. The most frequent choice for the first prescribed medicine had been a β-blocker (33.6% of cases) followed by ACE-inhibitors (27.7%) and Ca-antagonists (24.5%). For 16.7% of the patients the antihypertensive treatment was launched by the prescription of several drugs.

### Associated clinical conditions (ACC): self-reported data

Consistent with the study group formation based on diagnosis of HTN, 79% of participants (81% of women, 76% of men) had complications in cardiovascular system. Among the Estonian HTN patients recruited to the HYPEST study (n = 1,007), 119 individuals had experienced myocardial infarction (MI), 53 subjects had suffered from stroke and 120 had also diabetes (Table 5). Notably, the onset and prevalence of several clinical conditions showed considerable sex-biased differences (Table 5, 6). The first MI had occurred an average ~ 5 years earlier in male patients compared to females (51.4 ± 8.7 *vs*. 55.5 ± 8.0 years, respectively; *P *= 0.02). In total, the incidence of MI among hypertensive men was more than twice as high as in women (21.1% *vs*. 8.9%; *P *< 0.0001). This may be related to earlier onset of hypertension in men in general (Table 4). A trend of association was observed between earlier onset of HTN and increased number of documented MI incidences per patient (Pearson's correlation r = -0.12; *P *< 0.01). The diagnosis of diabetes showed similar trend of earlier onset in male HTN patients (47.8 ± 13.0 *vs*. 52.4 ± 10.8 years; *P *= 0.07), whereas no differences among sexes were estimated in disease prevalence (women: 14.6%, men 12.9%). Hypertensive women suffered significantly more from heart arrhythmia (76.7 *vs*. 61.0%; *P *< 0.0001). No gender effect was detected for the age of incidence of the first stroke as well as for the frequency of stroke (women: 6.0%, men 7.1%; Table 5, 6).

**Table 5:** Characteristics of associated clinical conditions (ACC) among Estonian hypertension patients

	Male			Female			
		
Parameter	n	Mean ± SD (95% CI)	Median	n	Mean ± SD (95% CI)	Median	*P*-value^a^ for gender comparison
Age of first stroke (y)	23	56.2 ± 10.3 (51.8-60.7)	53	30	57.2 ± 8.2 (54.1-60.3)	56	0.41
Age of first infarction (y)	70	51.4 ± 8.7 (49.4-53.5)	51	49	55.5 ± 8.0 (53.2-57.8)	56	0.02
Age of beginning of diabetes (y)	38	47.8 ± 13.0 (43.5-52.1)	50	82	52.4 ± 10.8 (50.0-54.8)	54.5	0.07
Duration of diabetes (y)	38	9.1 ± 10.9 (5.5-12.7)	5	82	8.0 ± 7.6 (6.3-9.6)	5.3	0.99

^a ^Mann-Whitney U-test; n - number of patients; SD - standard deviation; CI - Confidence Interval.

**Table 6:** Frequencies of self-reported clinical conditions profile in men and women

Variable	Men (%)	Women (%)	*P-*value^a^
*Diseases of cardiovascular system*			
Myocardial infarction	21.1	8.9	**< 0.0001**
Stroke	7.1	6.0	0.50
Ishaemic heart disease	29.2	33.3	0.21
Heart arrhythmia	61.0	76.7	**< 0.0001**
Other diseases of the circulatory system	15.6	19.4	0.40
*Diseases of genitourinary system*			
Glomerular diseases	0.7	1.6	0.49
Renal tubulo-intestinal diseases	2.8	16.8	**< 0.0001**
Renal failure	8.9	2.4	0.0001
Urolithiasis	8.5	9.3	0.79
Other disorders of kidney and ureter	5.2	9.0	0.06
Other diseases of urinary system	0.3	1.3	0.25
*Endocrine, nutritional and metabolic diseases*			
Diabetes	12.9	14.6	0.50
Thyroid disease	4.2	22.1	**< 0.0001**
Hyperthyroidism	1.9	13.9	**< 0.0001**
Hypothyroidism	2.2	8.0	**< 0.0001**
Hyperlipidaemia	30.1	46.1	**< 0.0001**
Other endocrine and metabolic diseases	2.5	0.7	0.17
Diseases of the digestive system	27.9	21.6	0.20
*Other complications*			
Asthma	5.6	7.1	0.42
Diseases of the musculoskeletal system and connective tissue	33.3	42.9	0.08
Neoplasms	6.5	11.4	0.15
Diseases of nervous system	8.2	11.7	0.38
Mental behavioural disorders	2.5	3.3	0.76
Diseases of the respiratory system	13.1	10.3	0.39
Diseases of the skin and subcutaneous tissue	1.6	2.6	0.73
Other diseases	11.5	22.0	0.02

^a ^Gender comparison, probability by Fisher's Exact test. Highly significant *P *-values (< 0.0001) have been indicated in bold.

Among other diseases, the HYPEST hypertensive men experienced 3-fold higher frequency of renal failure (women: 2.4%, men 8.9%; *P *< 0.0001; Table 6), whereas hypertensive women suffered significantly more (*P *< 0.0001) from renal tubulo-intestinal diseases (16.8% *vs*. 2.8%), thyroid diseases (22.1% *vs*. 4.2%) and hyperlipidemia (46.1% *vs*. 30.1%).

### Serum biomarkers

Majority of the HYPEST HTN patients (n = 756; 272 men/484 women) provided blood samples for the analyses of 11 serum biomarkers (detailed in Methods; Table 7). Consistent with disturbances in their cardiovascular system, HTN patients irrespective of sex exhibited elevated total cholesterol (mean value for men 5.5 g/l and for women 5.6 g/l) and LDL-cholesterol (mean value for men 3.7 g/l and for women 3.9 g/l) levels compared to normal laboratory reference range (< 5.0 g/l and < 3.0 g/l for total and LDL-cholesterol, respectively). For seven serum biomarkers the distribution of measured concentrations was statistically different between genders (*P*≤0.01). Urea, uric acid, creatinin and albumin levels were higher in men compared to women. Conversely, women exhibited higher concentration of K^+^, total cholesterol and HDL-cholesterol levels.

**Table 7:** The profile of serum biomarkers

	Male (n = 272)	Female (n = 484)		
Serum biomarker	Mean ± SD (95% CI)	Mean ± SD (95% CI)	Reference values^a^	*P*-value for gender comparison^b^
Na+ (mmol/l)	140.4 ± 2.2 (140.1-140.7)	140.3 ± 4.5 (139.9-140.7)	136-145	0.29
K+ (mmol/L)	4.3 ± 0.4 (4.3-4.4)	4.7 ± 6.1 (4.2-5.3)	3.5-5.1	0.01
Urea (mmol/L)	6.1 ± 2.0 (5.8-6.3)	5.7 ± 1.6 (5.5-5.8)	≤65 yrs < 8.3 > 65 yrs < 11.9	**0.009**
Creatinine (μmol/L)	88.5 ± 35.8 (84.2-92.7)	68.4 ± 29.2 (65.7-71.0)	M 62-106 F 44-80	**< 0.0001**
Uric Acid (μmol/L)	391.6 ± 84.0 (381.5-401.6)	317.8 ± 81.1 (310.6-325.1)	M 202-417 F 143-339	**< 0.0001**
Albumin (g/L)	44.6 ± 3.4 (44.2-45.0)	43.7 ± 2.8 (43.4-43.9)	35.0-52.0	**< 0.0001**
Cholesterol (g/l)	5.5 ± 1.1 (5.4-5.6)	5.6 ± 1.2 (5.6-5.9)	< 5.0	**0.007**
Triglycerides (mmol/L)	1.9 ± 1.9 (1.7-2.1)	1.7 ± 1.5 (1.6-1.9)	< 2.0	0.23
HDL-Choles-terol (g/l)	1.4 ± 0.4 (1.3-1.4)	1.6 ± 0.4 (1.5-1.6)	> 1.0	**< 0.0001**
LDL-Choles-terol (g/l)	3.7 ± 1.0 (3.6-3.8)	3.9 ± 1.0 (3.8-3.9)	< 3.0	0.08
C-reactive Protein (mg/L)	3.8 ± 7.8 (2.7-4.8)	3.3 ± 5.0 (2.8-3.8)	< 5.0	0.92

^a^http://www.kliinikum.ee/yhendlabor/index.php?option=com_content&view=article&id=48&Itemid=37; ^b ^Mann-Whitney U test; M - male; F - female; SD - standard deviation; CI - Confidence Interval; yrs - years; significant *P*-values (< 0.01) have been indicated in bold.

### Characteristics of life style risk factors

The analysis of current and retrospective lifestyle factors of the enrolled Estonian HTN patients was performed based on self-reported data. In general, the hypertensive study participants appear to be knowledgeable in monitoring their everyday food content and physical activities, as well as in restricting alcohol consumption and smoking (Table 8). Still, hypertensive women tend to follow healthier food, drinking and smoking habits (*P *< 0.0001), whereas male patients invest more to regular exercise (*P *= 0.0091). The proportion of never-smokers (non-smokers at diagnosis of HTN excluding previous smokers) was substantially higher in women (75.5% *vs*. 24.9%) and there were more smokers among men at the diagnosis of hypertension (36.1% vs. 10.8%; *P *< 0.0001) and at the recruitment (19.6% vs. 8.6%, *P *< 0.0001) (Table 8). Notably, 69.2% of Estonian HTN patients reported to have experienced regular stress.

**Table 8:** Gender differences of life style factors HYPEST hypertensive patients

Self-reported lifestyle factor	Male (%)	Female (%)	*P*-value for gender comparison^b^
*Status at recruitment*			
Current smoking	19.6	8.6	**< 0.0001**
Alcohol consumption more than once a week	22.1	2.1	**< 0.0001**
No current alcohol drinking	9.7	25.2	**< 0.0001**
Follows low caloricity	25.0	46.7	**< 0.0001**
Follows low fat content	54.4	74.8	**< 0.0001**
Follows low salt content	48.6	61.5	**< 0.0001**
Follows low sugar content	45.3	57.1	0.0004
Special diets	5.0	6.2	0.31
Physical loading at work (medium/high)	32.6	28.2	0.32
Physical activity at leisure time (> 2 times/week)	62.0	55.0	0.009
*Retrospective data*			
Smokers at diagnosis of hypertension	36.1	10.8	**< 0.0001**
Non-smokers at diagnosis of hypertension	44.4	82.4	
Previous smokers (at diagnosis)	19.5	6.9	
Regular sport in childhood	73.0	50.4	**< 0.0001**
Intensive sports in childhood	27.6	13.1	**< 0.0001**
No or occasional stress^a^	30.8	30.8	1.0
Expose to regular stress^a^	69.2	69.2	1.0
Periods of malnourishment^a^	30.0	36.1	0.06

^a ^during lifetime; ^b ^Fisher's Exact test. Highly significant *p*-values (< 0.0001) have been indicated in bold.

**Figure 2:**
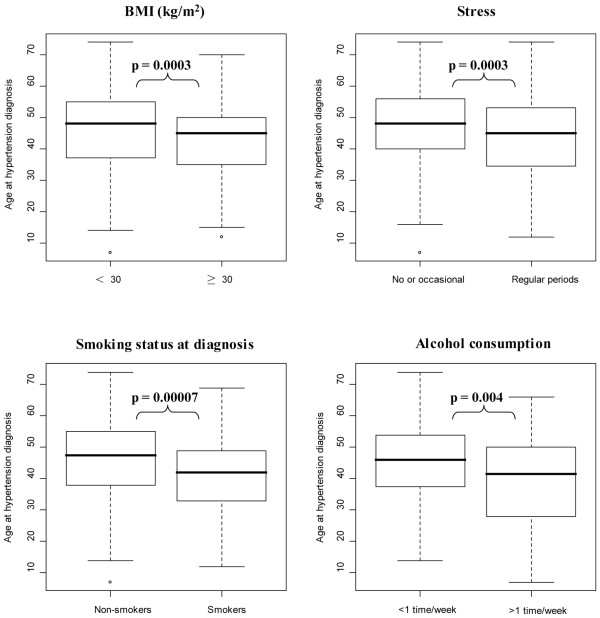
**Significant correlations (Mann-Whitney U-test) between the age at the onset of hypertension (HTN) and self-reported lifestyle risk factors**. The epidemiological questionnaires documented lifestyle factors at the recruitment, including also retrospective data for the lifetime. Smokers and non-smokers were defined based on smoking status at the diagnosis of HTN. Obesity was defined as BMI ≥ 30 kg/m^2^. Life-long alcohol consumption habits were classified as: less than once a week (no or restricted alcohol intake) and more than once a week. Stress factor was divided into subgroups exhibiting (i) regular or (ii) no/occasional stress. The first group included individuals who reported regular stress during recruitment or had experienced regular stress period during lifetime. The second group consisted of individuals who had never experienced stress or had reported only occasional stress episode during their life.

We attempted to estimate the role of individual lifestyle risk factors (smoking, BMI, stress and alcohol consumption) on the age of onset of hypertension (Figure 2). All these risk factors were shown to significantly affect the age of hypertension onset (*P *< 0.005). The most significant effect was detected for smoking, which strongly enhanced the development of HTN. Among non/previous smokers (smoking status at the diagnosis of HTN), the age of the diagnosis of HTN (47.5 ± 13.8 years) was approximately 5 years later (*P *= 0.00007) compared to the subgroup of regular smokers (42.0 ± 12.3 years).

## Discussion

In Estonia, cardiovascular diseases (including hypertension) are the most common cause of death both in men and women [3]. Despite the fact that the prevalence of cardiovascular diseases as a cause for loss of life years has decreased significantly during the past decade in Estonia, it is still nearly two times higher than in other European countries [17]. A clear need exists for a more systematic understanding of the epidemiology, diagnosis, and management of hypertension in Estonia. We established HYPEST sample collection, which provides a good resource for studying genetic-epidemiological component of HTN in Estonian population. This report is focused on the comparative analysis of disease and lifestyle profiles of Estonian male and female hypertensive patients, who were enrolled during the HYPEST study from 2004 to 2007. Patients were recruited in two main healthcare centers covering major part of Estonia. In most published epidemiological studies BP measurements were conducted during a single visit [1]. In HYPEST study, the diagnosis of hypertension was defined by cardiologist and relies on several consecutive BP measurements. The overall fraction of female patients among invited (~ 59%) and recruited (61%) study participants was higher compared to male hypertensives. This bias was introduced due to higher proportion of women with available clinically diagnosed essential hypertension records in participating centers. Also, the age distribution is different between genders; with much less men belonging to the age group 60+. The lower proportion of elderly male patients could be influenced by the fact that, in Estonia, the mean life expectancy in men is considerably shorter than in women (67.1 years in men vs 78.7 years in women), which makes it difficult to include older men into the study [3].

Men and women differ in the pathophysiology, risks and treatment of essential hypertension. The prevalence of hypertension increases with age and is higher among men than women before the age of 55 years, but slightly higher among women thereafter [18]. Similar trend was detected in current study, where the age of hypertension diagnosis in male patients was around 5 years earlier compared to female cases. The higher age of female population could reflect, in part, the protective hormone effect before menopause, which could postpone the onset of hypertension [10,19]. The elevated blood pressure is a high risk factor for later cardiovascular and renal diseases and therefore it was not surprising that patients had markedly higher prevalence of cardiovascular diseases compared to general population [20]. The most common self-reported cardiovascular problem for both sexes was heart arrhythmia, followed by ischemic heart disease. The observed earlier occurrence of the first myocardial infarction as well as higher number of incidences in male patients is probably related to the earlier onset of hypertension in men. This is consistent with previous studies done in Estonia, showing that in case of men, cardiovascular diseases occur earlier compared to women [21,22]. Problems with cardiovascular system were also reflected in serum biomarker analysis, showing increased total and LDL-cholesterol levels among patients compared to normal laboratory reference range. In general, women seem to be keener to monitor their health compared to men, which is reflected in higher number of reported incidences of other diseases in the self-reported questionnaire.

It is widely known that lifestyle behaviors such as reducing weight and alcohol intake, quitting smoking, increasing physical activity and eating a healthy diet are related to the prevention and control of elevated blood pressure [22,23]. Current study clearly demonstrates that reducing the conventional lifestyle risk factors could delay the age of hypertension onset. This effect was most significant with smoking status, which is concordant with recent study done in Japanese population [12]. Another generally accepted risk factor for increased blood pressure is elevated BMI [8,11]. One limitation of the current study is unavailable BMI data at the time of HTN diagnosis. Thus, to assess the effect of excess weight on the onset of hypertension, we used in our analysis stringent criteria to subdivide HYPEST HTN patients based on BMI data collected at the recruitment (obese ≥ 30 kg/m^2 ^and non-obese < 30 kg/m^2^). In general, study participants appeared to be aware of the risk factors and seemed to consciously reduce them in their everyday life. For example, among hypertensive patients the number of current smokers is two times lower compared to general Estonian population [24,25]. Furthermore, almost half of the male patients who reported current smoking habit during the diagnosis of hypertension had quit smoking after diagnosis.

Unfortunately excessive weight gain shows increasing trend in general Estonian population, which is also reflected in HYPEST data [3,24,26]. The majority of HYPEST patients are overweight (BMI ≥ 25 kg/m^2^) and this proportion is approximately 20% higher than average in 40-70 years old Estonian people [27]. Observed mean values for BMI among the recruited HYPEST patients were higher (29.8 ± 5.0 kg/m^2 ^for males; 30.4 ± 5.5 kg/m^2 ^for females) than respective means of age and gender in general Estonian population (27.8 ± 4.2 kg/m^2 ^for males; and 28.4 ± 5.6 kg/m^2 ^for females) in age group 50-60 years [26]. Probably problems with overweight among HTN patients have encouraged them to pay more attention to the everyday diet as well on the physical activity. However, although dietary habits in Estonia have improved during the last decade, one third of all participated patients (33.72%) notified periods of malnourishment during their lifetime. This could be explained by the fact that most of study participants belong to age groups where they could have been influenced by the period of Second World War. Maternal diet during the pregnancy and/or poor nutrition in early childhood could both influence the development of cardiovascular disease (including hypertension) in adulthood [28]. Finally, the hypertensive patients, who participated in the HYPEST study show awareness of their health conditions and have been responsive in adopting healthy lifestyle recommendations given by their physicians.

The present study has certain limitations that should be taken into account for interpretation of the results. First, the retrospective self-reported epidemiological data may be biased and tend to exaggerate the occurrence of events. Second, the non-equal distribution of men/women patients as well as the age distribution among sexes might affect the gender-related differences and bias the estimate. In addition, the study sample is only representative of volunteers and findings may therefore not be generalized to all Estonian hypertension patients. Finally, the retrospective BP data extracted from patient's clinical records were measured with different clinical settings (investigators, devices, laboratories) and thus, certain undefined measurement variability could have been introduced into the estimates. Despite these limitations, the present study gives a profile of Estonian hypertensive patients by analyzing considerably larger number of subjects than earlier investigations [6,7,24].

## Conclusions

In summary, the current study characterizes the profile of HTN patients in Estonia. We compared disease and lifestyle parameters of male and female hypertensive patients recruited for the HYPEST study. Our findings are in agreement with published studies, which have revealed gender-specific differences in (i) the age at HTN onset, (ii) HTN risk factors affecting males and females, and (iii) secondary complications of the disease. HYPEST sample collection, combining the clinically diagnosed HTN cases (n = 1,007) and population-based controls across Estonia (n = 959) is a valuable source for further epidemiological as well as genetic studies of hypertension [29-37].

## Competing interests

The authors declare that they have no competing interests.

## Authors' contributions

ML initiated and directed the research, and EO, MV and ML designed jointly the study. All authors participated in the recruitment of study subjects and collection of epidemiological data. GV, EO, MV, MR and TU contributed to the collection of clinical data. GV, EO and ML designed, and GV performed the statistical analysis. EO, GV, PJ and ML drafted the first manuscript and MV, MP, MR, KT and TU revised the interpretation of the data and results, and contributed to the preparation of the final manuscript text. All authors read and approved the final manuscript.

## Pre-publication history

The pre-publication history for this paper can be accessed here:

http://www.biomedcentral.com/1471-2261/11/55/prepub
